# Diagnostic Utility of Cartridge-Based Nucleic Acid Amplification Test (CBNAAT) on Induced Sputum Versus Gastric Aspirate Samples for the Diagnosis of Paediatric Pulmonary Tuberculosis

**DOI:** 10.7759/cureus.47246

**Published:** 2023-10-18

**Authors:** Dipti Mishra, Amit Singh, Rajesh K Yadav, Mudit Verma

**Affiliations:** 1 Microbiology, Uttar Pradesh University of Medical Sciences, Etawah, IND; 2 Pediatrics, Uttar Pradesh University of Medical Sciences, Etawah, IND; 3 Community Medicine, Uttar Pradesh University of Medical Sciences, Etawah, IND

**Keywords:** paediatric tb, induced sputum, gastric aspirate, rifampicin resistance, zn staining, mgit, genexpert

## Abstract

Background: Tuberculosis (TB) in children is neglected, mainly due to a lack of sensitive diagnostic tools. Paediatric TB is now a global priority. More paediatric TB cases are being recorded as a result of the introduction of Xpert® Mycobacterium tuberculosis (MTB)/rifampicin (RIF) (Cepheid Inc., Sunnyvale, USA). This study was undertaken to evaluate the performance of Xpert MTB/RIF in the diagnosis of pulmonary TB in children.

Methods: We recruited 70 paediatric patients with probable pulmonary TB and their gastric aspirate (GA), and induced sputum (IS) samples were collected between January 2021 and June 2022 in Saifai, Etawah, Uttar Pradesh, at the Microbiology Department of the Uttar Pradesh University of Medical Sciences (U.P.U.M.S.). All samples were subjected to smear examination, Bacterial Activation of Continuous Temperature and Environmental Control - Mycobacterial Growth Indicator Tube (BACTEC-MGIT) culture, and Xpert MTB/RIF.

Results: The specimens included 70 GAs and 70 IS samples. The total number of specimens were 140 and we collected GA as well as IS from each of the patient enrolled in the study. When compared to microscopy, GeneXpert provides a quicker and earlier detection of paediatric TB. The sensitivity of the cartridge-based nucleic acid amplification test (CBNAAT) against mycobacterial growth indicator tube (MGIT) was 75.0% for GA samples and 63.64% for IS samples.

Conclusion: Paediatric TB, owing to its paucibacillary nature and difficulty in the collection of samples, makes the diagnosis difficult by conventional methods. Our study shows that smear and culture yield in GA samples are superior to those of IS samples and the sensitivity of Xpert MTB/RIF assay is also significantly different in GA and IS samples, but a combination of GA and IS yielded the best results.

## Introduction

As per the World Health Organization’s (WHO) Global Tuberculosis Report 2021, an estimated 10.6 million people were infected with tuberculosis (TB), with which 6 million men, 3.4 million women, and 1.2 million children were affected [1]. The burden of the disease varies, from fewer than five to more than 500 new cases per 100,000 population per year, with the global average being around 130 [2].

For children of all ages across the world, particularly young children, TB continues to be a leading cause of morbidity and mortality from infectious illnesses. The Global T.B. Report 2021 estimates that every year, 3.06 lakh children (0-14 years of age) contract TB, making up around 11% of all estimated TB cases reported to the NTEP (National Tuberculosis Elimination Program). Paediatric TB is an enormous issue in India, accounting for around 31% of the worldwide burden. Both globally and domestically, the mortality of paediatric DR (drug-resistant)-TB data continues to be a problem. Just 12,200 (11%) of the worldwide goal of 1,15,000 were reached in terms of mortality in the paediatric age group as a result of drug-resistant TB [1].

Due to its atypical presentation compared to the adult population, paediatric TB is a neglected disease. Typically, children are unable to expectorate or generate modest amounts of sputum. Sputum smear microscopy can only identify a small number of bacilli since they are rare in respiratory secretions. The following alternative samples are available: laryngeal swabs, bronchioalveolar lavage (BAL), induced sputum (IS), nasopharyngeal aspirate, etc. These samples have varied sensitivity and specificity depending on the diagnostic test employed. According to reports, gastric aspirate (GA) samples had the highest (40-92%) detection rate, depending on the sensitivity of the laboratory test used. Detection rates for other samples were from 4-43% for BAL, 24-30% for nasopharyngeal aspiration, 27-63% for a laryngeal swab, and 20-30% when utilizing IS [3]. Thus, GA and IS are important samples for the diagnosis of paediatric pulmonary TB. Smear microscopy by Ziehl-Neelsen (ZN) staining for acid-fast bacilli is a rapid and inexpensive method for the diagnosis of TB but lacks sensitivity [4].

The best method for studying mycobacteria is by culture, although this process takes time (between two and six weeks) and involves both technical expertise and a suitable laboratory setup. An automated liquid culture device for mycobacterial identification is the BD BACTEC™ Mycobacteria Growth Indicator Tube (MGIT™) system (Becton Dickinson, Buenos Aires, Argentina). Compared to traditional solid culture on the Lowenstein-Jensen (LJ) medium, it is created and optimized for the quick identification of mycobacteria from clinical specimens [5].

The Xpert® Mycobacterium tuberculosis (MTB)/rifampicin (RIF) assay (Cepheid Inc., Sunnyvale, USA) is an automated nucleic acid amplification test for the simultaneous detection of Mycobacterium TB complex (MTBC) and its resistance to RIF directly from clinical samples. The test may be performed on chemically inactivated specimens and does not need sample preparation and results are available in 2 hours. Thus, it is simple, less time-consuming, and does not need specialized technological expertise or biosafety criteria. It was the sole fast molecular test first suggested by WHO in 2010 for pulmonary TB diagnosis in adults. It has also been suggested for usage in children since 2013 and also can be used to identify extrapulmonary TB [6].

This study aimed to assess the diagnostic utility of Xpert assay on GA versus IS samples for diagnosing paediatric pulmonary TB along with that of conventional methods.

## Materials and methods

Study design and subject recruitment

The study was conducted between January 2021 and June 2022 in the Department of Microbiology at Uttar Pradesh University of Medical Sciences, Saifai, Etawah, Uttar Pradesh. A total of 92 children, aged 6 months to 14 years with suspected pulmonary TB symptoms were enrolled. Twenty-two children were excluded due to insufficient sample volume and whom ATT (antitubercular therapy) had already started.

Any children with a cough and fever lasting more than two weeks with no improvement after taking amoxicillin for seven to ten days, recent unexplained weight loss, or a history of contact with a patient with TB in the three months prior were included. Our exclusion criteria included children older than 15 years of age, patients, already receiving ATT, samples received without clinical history, patients whose guardians had not given consent for enrolling in the study, and an insufficient volume of specimens.

Gastric aspiration and IS collection were performed on all children with suspected intra-thoracic TB. Children were kept fast for at least four hours to ensure accurate test results before undergoing GA and IS sample collection. After explaining the procedure and obtaining informed consent for enrolment in the study and, for TB testing, was obtained from a parent, an appropriately sized feeding tube (10-12G) was inserted through one nostril till it reached the stomach.

The position of the tube was checked by the insufflation of air into the stomach. The contents of the stomach were aspirated completely and kept in a sterile container. The usual volume collected was around 10 millilitres (mL). Samples were transported to the microbiology laboratory for further processing within 1-2 hours. At the same sitting, gastric aspiration was performed first, and then, IS was performed after a 30-minute gap. To IS, the child was first primed with two puffs of salbutamol administered using a metered dosage inhaler (MDI Asthalin, 100 g/puff, Cipla, India) and spacer, which was followed by 15-20 minutes of nebulization with 3 mL of 3% saline. All samples received unique Lab ID numbers and were transmitted to the lab. Samples were processed on the same day for the acid-fast bacilli (AFB) smear, Bacterial Activation of Continuous Temperature and Environmental Control - Mycobacterial Growth Indicator Tube (BACTEC-MGIT) culture, and cartridge-based nucleic acid amplification test (CBNAAT) [7].

Ethical considerations

The study was approved by the Institutional Ethics Committee of Uttar Pradesh University of Medical Sciences (U.P.U.M.S.), Saifai, Etawah, UP. Each child's parents or guardians provided their written consent after receiving adequate information.

Laboratory methods

All the specimens were processed with Ziehl-Neelsen (ZN) staining, Xpert, and cultured on Middlebrook 7H9 liquid media (Becton Dickinson, USA). At a level III biosafety laboratory, standard operating procedures were followed for concentration, decontamination using the N-Acetyl-L-Cysteine-Sodium Hydroxide (NALC-NaOH) technique, and processing of specimens using the Xpert MTB/RIF test and liquid culture. In two hours, the GeneXpert Dx system produced the results for Xpert [8,9].

The sample was divided into two parts. After centrifuging the first portion, two drops of the sample pellet (or around 100 µl) were used to prepare smears and stain them using the ZN procedure. The slides that showed pink-coloured AFB were taken as smear‑positive [10]. For GeneXpert assay processing, the sample reagent buffer containing sodium hydroxide (NaOH) and isopropanol was added in the ratio of 3:1 (total 2 mL) and incubated for 15 min at room temperature. Thereafter, 2 mL of the prepared sample was added to the Xpert MTB/RIF cartridge, which also contained a wash buffer and lyophilized reagents for deoxyribonucleic acid (DNA) extraction and polymerase chain reaction (PCR) amplification. According to the manufacturer's instructions, the cartridge was loaded into the Xpert MTB/RIF device after being properly mixed. The device automatically executes specimen mixing, sonication of the internal control (spores) and mycobacterial bacilli, DNA release, and reagent mixing for PCR. This is followed by a hemi-nested real-time PCR amplification, target detection by five-colour fluorescence molecular beacon probes, and internal control in situ. In two hours, results are presented and categorised as follows: semi-quantified bacillary burden high, medium, or low; RIF sensitivity or resistance; and *M. tuberculosis *positive or negative. The patient's data was matched to the samples for examination only after the Xpert MTB/RIF evaluation was complete [11].

Lastly, the second portion of the sample was treated using the N acetyl L cysteine sodium hydroxide (NALC NaOH) procedure, grown on MGIT media, and incubated in the MGIT BACTEC 960 liquid culture system. As per the manufacturer's recommendations, ZN staining and culture on 5% sheep blood agar were carried out immediately from the tube when a sample was marked positive to check for bacterial contamination. Before being identified as negative by the system, all of the tubes were incubated for 42 days. A rapid immunochromatography test (ICT) kit was used to identify Mycobacterium tuberculosis protein (MPT) 64 antigen in tubes that had tested positive for AFB by smear microscopy. ICT-negative tubes were categorized as nontuberculous Mycobacterium (NTM) species, whereas ICT-positive tubes were designated as the MTB complex [12].

Statistical analysis

The sensitivity, specificity, positive predictive value (PPV), and negative predictive value (NPV) of the Xpert MTB/RIF assay were calculated using liquid culture (MGIT) results as the reference standard. In this study, a p-value of 0.05 or lower was considered statistically significant. Data thus collected were encoded in a Microsoft Excel worksheet (Microsoft® Corp., Redmond, WA) and analysed using Statistical Package for the Social Sciences (IBM SPSS Statistics for Windows, IBM Corp., Version 25, Armonk, NY).

## Results

This study was conducted at U.P.U.M.S, Saifai at the Department of Microbiology. Only those children were included from whom both GA and IS samples were collected. For this purpose, 70 children aged 6 months to 14 years presenting with symptoms of TB and fulfilling inclusion and exclusion criteria were enrolled in the study.

Out of the 70 samples of GA, 15 (21.4%) were positive by Xpert assay, six (8.57%) were positive for AFB in smear microscopy, and 16 (22.9%) were obtained to be culture positive. Out of 16 culture-positive GA samples, CBNAAT was able to detect 12 cases. Out of 10 culture-positive cases, which were negative in the smear, CBNAAT diagnosed only seven cases and the remaining three were negative by CBNAAT. Moreover, three samples were smeared‑ and culture‑negative was detected positive by GeneXpert.

**Figure 1:**
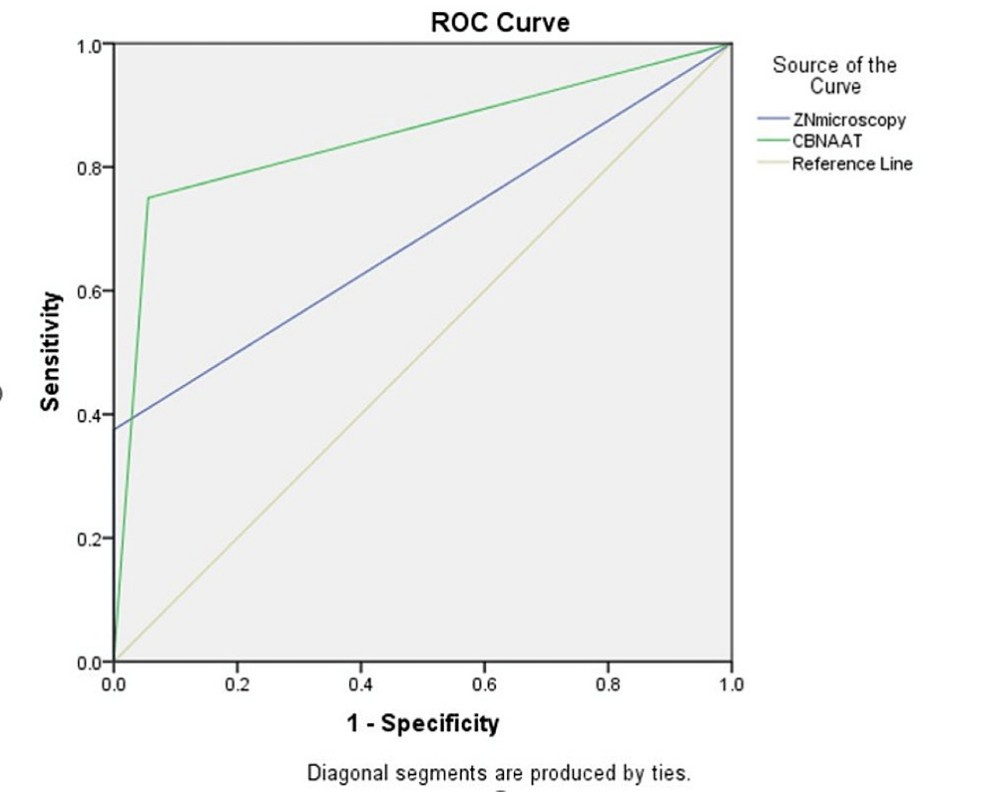
Receiver operating characteristic curve (RoC) for comparison of ZN Microscopy, CBNAAT with MGIT in gastric aspirate ZN - Ziehl-Neelsen, CBNAAT - cartridge-based nucleic acid amplification test, MGIT - mycobacterial growth indicator tube

Table 1 shows the comparison of ZN Microscopy, and CBNAAT with MGIT in GA using the receiver operating characteristic (RoC) curve (Figure 1), where ZN Microscopy with MGIT shows the area of the curve is 0.688, p-value = 0.023<0.05 with 95% CI 0.517 to 0.858, which is statistically significant with a sensitivity of 37.5%, specificity 100.0%, PPV 100.0%, NPV 84.37%, and accuracy is 85.71% (Table 2), and in CBNAAT with MGIT shows the area of the curve is 0.847, p-value = 0.001<0.01 with 95% CI 0.715 to 0.979, which is the highly statistical significance with the sensitivity 75.0%, specificity 94.4%, PPV 80.0%, NPV 92.7% and accuracy is 90.0% (Table 2).

**Table 1:** Comparison of ZN Microscopy, CBNAAT with MGIT in gastric aspirate using receiver operating characteristic curve (RoC) ZN - Ziehl-Neelsen, CBNAAT - cartridge-based nucleic acid amplification test, MGIT - mycobacterial growth indicator tube, CI - confidence interval, LB - lower bound, UB - upper bound ** Highly statistical significance at p < 0.01, * Significant at p < 0.05 and # No statistical significance at p > 0.05

Test Result Variable(s)	Area Under Curve	Standard Error	p-value	95% CI	
				LB	UB
ZN Microscopy	.688	.087	0.023*	.517	.858
CBNAAT	.847	.067	<0.001 **	.715	.979

**Table 2:** Comparison of ZN Microscopy, CBNAAT with MGIT in GA and IS ZN - Ziehl-Neelsen, CBNAAT - cartridge-based nucleic acid amplification test, MGIT - mycobacterial growth indicator tube, PPV - positive predictive value, NPV - negative predictive value, GA - gastric aspirate, IS - induced sputum

S. No.		Variables	GA (%)	IS (%)
1.	ZN Microscopy	Sensitivity	37.5	16.7
		Specificity	100.0	100.0
		PPV	100.0	100.0
		NPV	84.37	85.3
		Accuracy	85.71	85.7
2.	CBNAAT	Sensitivity	75.0	63.64
		Specificity	94.4	96.61
		PPV	80.0	77.77
		NPV	92.7	93.44
		Accuracy	90.0	91.42

Out of the 70 samples of IS, nine (12.9%) were positive by Xpert assay, two (2.9%) were positive for AFB in smear microscopy, and 11 (15.71%) were obtained to be culture positive.

Out of 11 culture-positive IS samples, CBNAAT was able to detect seven cases. Out of nine culture-positive cases, which were negative in the smear, CBNAAT diagnosed only five cases and the remaining four were negative by CBNAAT. Moreover, two samples that were smear and culture‑negative were detected positive by GeneXpert.

**Figure 2:**
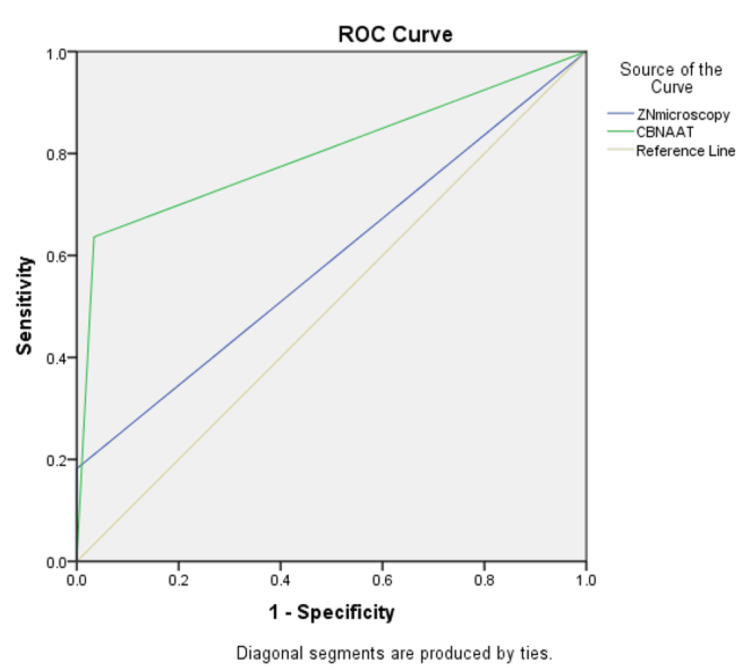
Receiver operating characteristic curve (RoC) for comparison of ZN Microscopy, CBNAAT with MGIT in induced sputum ZN - Ziehl-Neelsen, CBNAAT - cartridge-based nucleic acid amplification test, MGIT - mycobacterial growth indicator tube

Table 3 shows the comparison of ZN Microscopy and CBNAAT with MGIT in IS using the RoC curve (Figure 2), where ZN Microscopy with MGIT shows the area of the curve is 0.591, p-value = 0.341>0.05 with 95% CI 0.389 to 0.793, which is no statistically significant with the sensitivity 16.7%, specificity 100.0%, PPV 100.0%, NPV 85.3% and accuracy is 85.7% (Table 2), and in CBNAAT with MGIT shows the area of the curve is 0.801, p-value = 0.002<0.01 with 95% CI 0.623 to 0.979, which is the highly statistical significance with the sensitivity is 63.64%, specificity 96.6%, PPV 77.8%, NPV 93.4%, and accuracy is 91.4% (Table 2).

**Table 3:** Comparison of ZN Microscopy, CBNAAT with MGIT in induced sputum using receiver operating characteristic curve (RoC) ZN - Ziehl-Neelsen, CBNAAT - cartridge-based nucleic acid amplification test, MGIT - mycobacterial growth indicator tube, CI - confidence interval ** Highly significant at p < 0.01 and # No statistical significance at p > 0.05

Test Result Variable(s)	Area	Standard Error	p-value	95% CI	
				LB	UB
ZN Microscopy	.591	.103	0.341 #	0.389	0.793
CBNAAT	.801	.091	0.002 **	0.623	0.979

Overall, in samples (IS + GA) that were positive either in smear and/or culture (27/140), the sensitivity of Xpert MTB/RIF assay was slightly lower (70.37%, 19/27) as compared to bacteriological examination (Table 4) but it also detected 4.42% (5/113) additional cases over the culture giving an overall sensitivity of 17.14% (24/140). The performance of Xpert MTB/RIF was 87.5% (7/8) on smear and culture-positive samples, as shown in (Table 4). If all culture-positive samples were taken together without considering the mycobacterial species, the sensitivity fell to 63.15% (12/19). So, a combination of one GA and one IS sample gave the best diagnostic utility.

**Table 4:** Performance of Xpert MTB/RIF assay in bacteriologically positive and bacteriologically negative samples (combination of gastric aspirates (n = 70) and induced sputum (n = 70)) collected from children with probable intrathoracic tuberculosis +ve - positive, -ve - negative, CBNAAT - cartridge-based nucleic acid amplification test, MTB/RIF - Mycobacterium tuberculosis/rifampicin

Bacteriological Criteria (N=140)	Sub-Criteria	No (%)	CBNAAT Results	
			+ve	-ve
Bacteriologically +ve (N=27;19.28%)	Smear +ve Culture +ve	8 (5.71%)	7 (87.5%)	1 (12.5%)
	Smear -ve Culture +ve	19 (13.57%)	12 (63.15%)	7 (36.84%)
	Smear +ve Culture -ve	0	0	0
Bacteriologically -ve (113; 80.71%)	Smear -ve Culture -ve	113	5 (4.42%)	108 (95.57%)

## Discussion

Due to the nonspecific presentation of paediatric pulmonary TB, it is still difficult to diagnose. In order to effectively treat the condition and stop the spread of infection, laboratory confirmation of TB is essential. Smear microscopy requires a bacterial load of 10^4^/mL organisms to classify a sample as positive, whereas GeneXpert requires 131 CFU/mL of bacilli to do so [13]. Similar to the results of prior research, in our study, GeneXpert was able to diagnose twice as many instances as ZN staining [14,15].

The majority of paediatric TB cases are smear-negative due to the typical paucibacillary presentation in children and their inability to produce sputum on demand. Children often cannot expectorate sputum or make minimal amounts of it. Sputum smear microscopy can not detect a small number of bacilli since they are very few in respiratory secretions.

So, in this study, IS and GA were used to investigate children with suspected PTB. The diagnostic performance of Xpert is comparable in IS and GA specimens. Depending on the sensitivity of the laboratory test used, GA samples have been found to have the highest detection rate (40-92%), whereas IS provides detection rates of 20-30% [5].

In this study, the diagnostic yield for microscopy, MGIT960, and CBNAAT were 8.57%, 22.9%, and 21.4% respectively for GA samples and the diagnostic yield for microscopy, MGIT960, and CBNAAT were 2.9%, 15.7%, and 12.9% respectively for IS samples. Sharma S et al. [5] in their study found that GA samples provide better diagnostic yield for the detection of paediatric pulmonary TB than IS samples. The findings show that diagnostic yield for different diagnostic modalities varies in different studies. Within the same study, microscopy was related to lower positivity rates while culture was associated with a higher positivity rate. This could be due to the fact that paediatric TB cases are paucibacillary in nature and culture helps to enhance the bacillary count substantially making it easier to detect.

In the present study, CBNAAT detected 10 positive cases in 64 smear-negative samples of GA and seven positive cases in 68 smear-negative samples of IS. Out of 16 culture-positive GA samples, CBNAAT was able to detect 12 cases, and out of 10 culture-positive cases, which were negative in the smear, CBNAAT diagnosed only seven cases and the remaining three were negative by CBNAAT. For IS samples CBNAAT detected seven positive cases in 68 smear-negative samples. Out of 11 culture-positive IS samples, CBNAAT was able to detect seven cases and out of nine culture-positive cases, which were negative in the smear, CBNAAT diagnosed only five positive cases and the remaining four were negative. CBNAAT detected three positive samples for GA and two positive samples for IS which were negative by both smear and MGIT.

Samples that were culture-positive and smear‑negative, flagged negative by the GeneXpert, these samples later showed the growth of NTM species. Results from the GeneXpert test that are positive but have negative results for the culture should be carefully analyzed and compared with the patient's medical history because such MGIT negative cases usually indicate the dead bacillary load in the samples and may point toward inactive infection, which can still be picked up by the GeneXpert because of the excretion of the leftover DNA from the deceased bacilli following the use of antitubercular drugs. Hence good clinical acumen is still needed to decide when to start ATT.

In the present study, the sensitivity, specificity, PPV, NPV, and accuracy of ZN Microscopy against MGIT were 37.5%, 100.0%, 100.0%, 84.37%, and 85.71% respectively for GA samples and sensitivity, specificity, PPV, NPV and accuracy of ZN Microscopy against MGIT was 16.7%, 100.0%, 100.0%, 85.3%, and 85.7% respectively for IS samples. The sensitivity and accuracy of microscopy for the GA sample are more than IS sample.

The sensitivity, specificity, PPV, NPV, and accuracy of CBNAAT against MGIT were 75.0%, 94.4%, 80.0%, 92.7%, and 90.0% respectively for GA samples. The sensitivity, specificity, PPV, NPV, and accuracy of CBNAAT against MGIT were 63.64%, 96.6%, 77.8%, 93.44%, and 91.42% respectively for IS samples. In our study, CBNAAT shows a higher sensitivity for GA (75.0%) than IS samples (63.64%). our results were in total concordance with the findings of Sekadde et al. [14]. Detjen A et al. [9] reported the sensitivity and specificity of CBNAAT against MGIT were 62% and 98% respectively for GA samples.

A combination of GAs and IS samples yielded a very high TB detection rate by Xpert MTB/RIF. The overall sensitivity of Xpert MTB/RIF on smear and culture-positive samples was 87.5%. In smear-negative and culture-positive samples the sensitivity of Xpert MTB/RIF was 63.15%. But in bacteriologically confirmed samples, as expected, the detection rate was higher (70.37%). Our results were in total concordance with the findings of Singh S et al. [7] reported sensitivity of Xpert MTB/RIF assay for smear and culture-positive GA and IS samples was 95.6% and the sensitivity of Xpert MTB/RIF assay on culture-positive but smear-negative samples was lower (62.5%).

The detection rate of RIF-resistant MTB among a total of 70 children was found to be two (2.9%). Various studies reported the resistance rate among paediatric TB to be 0.6% to 2.3%. The results match the percentages shown in other studies [13]. Lohiya Ayush et al. reported the resistance rate among children affected with TB to be 5.1% [16].

The limitation of the study is the low positive rate due to a very limited sample size. The study may produce better results if it is expanded to a larger sample size or is carried out as a multi-centric study.

## Conclusions

Paediatric TB, owing to its paucibacillary nature and difficulty in the collection of samples, makes the diagnosis difficult by conventional methods. Culture, although being the gold standard, is not used routinely due to delays in results. IS is a safe and well-tolerated technique and can be successfully performed even on infants. In children with suspected paediatric TB, GA remains the standard technique for microbiological diagnosis. However, taking into account the growing issue of resistance strains, IS should also be taken into consideration, at least in developing countries, as a supplemental tool to boost the diagnostic yield of paediatric TB. Our study shows that smear and culture yield in GA samples are superior to those of IS samples and the sensitivity of Xpert MTB/RIF assay is also significantly different in GA and IS samples, but a combination of GA and IS yielded the best results.
